# Exploring Genetic Drug Targets in Acne Vulgaris: A Comprehensive Proteome‐Wide Mendelian Randomization Study

**DOI:** 10.1111/jocd.16567

**Published:** 2024-09-19

**Authors:** Ruyi Ju, Yuou Ying, Qiujun Zhou, Yi Cao

**Affiliations:** ^1^ The First School of Clinical Medicine Zhejiang Chinese Medical University Hangzhou City Zhejiang Province China; ^2^ Zhejiang Chinese Medical University and the First Affiliated Hospital of Zhejiang Chinese Medical University Hangzhou City Zhejiang Province China

**Keywords:** acne vulgaris, drug target, genetics, inflammation, Mendelian randomization

## Abstract

**Background:**

Acne vulgaris presents a substantial clinical challenge due to its complex pathophysiology and significant impact on quality of life. Identification of novel therapeutic targets for acne using genetic tools can guide the development of more effective treatments.

**Methods:**

Utilizing a dataset comprising 35 559 Icelandic individuals, we performed proteomic analyses to quantify 4709 circulating proteins. We integrated these data with acne‐specific genome‐wide association studies (GWAS) encompassing 34 422 acne patients and 364 991 controls. Mendelian randomization (MR) analyses employed the TwoSampleMR tool and Summary‐data‐based Mendelian Randomization (SMR) to estimate the causal effects of identified proteins on acne risk. Colocalization analyses assessed the likelihood of shared genetic etiology between protein levels and acne using the “coloc” R package.

**Results:**

Our proteome‐wide MR analysis initially identified 128 proteins potentially associated with acne risk. Following multiple testing corrections using the Benjamini–Hochberg method, fatty acid synthase (FASN) and tissue inhibitor of metalloproteinases 4 (TIMP4) remained significantly associated with acne risk. FASN exhibited a protective effect against acne (OR = 0.768, 95% CI: 0.676–0.872, *p* = 4.685E‐05), while TIMP4 was associated with an increased risk (OR = 1.169, 95% CI: 1.103–1.241, *p* = 1.956E‐07). Colocalization analysis supported a shared genetic basis for these protein‐acne associations, with posterior probabilities indicating strong evidence of shared causal variants.

**Conclusion:**

Our findings highlight the utility of integrative genomic approaches in identifying potential therapeutic targets for acne. FASN and TIMP4, in particular, demonstrate strong potential as targets for therapeutic intervention, pending further validation through clinical research. These results offer a foundation for targeted acne treatment development, aligning with personalized medicine principles.

## 1 Introduction

Acne vulgaris is a prevalent chronic inflammatory dermatological condition predominantly affecting sebaceous glands in densely populated areas such as the face, back, and chest. It manifests clinically as a spectrum of lesions, including closed and open comedones, papules, pustules, nodules, and cysts. Progression of acne may result in skin pigmentation and scarring, severely affecting patients' psychological well‐being and quality of life [1]. Epidemiological data indicate that while acne primarily affects adolescents and young adults, it is not confined to these age groups [2, 3]. Approximately 85% of individuals globally experience acne at some point in their lives, ranking it among the most common skin disorders [4]. Acne's pathogenesis is multifaceted, involving genetic factors, increased sebum production, abnormal follicular keratinization, microbial proliferation (notably Propionibacterium acnes), and the immune system's inflammatory response [5, 6]. Although some cases of acne resolve spontaneously, untreated severe instances can cause enduring skin damage and psychosocial complications. Acne's onset is intricately linked to dietary habits, lifestyle choices, and psychological stress levels, posing significant challenges in management due to these factors' complex interplay [7]. Current therapeutic approaches, which include topical and systemic treatments, vary in effectiveness and may lead to adverse reactions in some patients. Therefore, the identification of novel therapeutic targets and the development of more efficacious treatments with fewer side effects remain critical objectives in acne research.

Mendelian randomization (MR) is a statistical technique that employs genetic variants as instrumental variables to evaluate the causal effects of specific exposure factors, such as protein levels, on disease risk [8]. MR has enriched our comprehension of pathophysiological mechanisms, especially valuable in contexts where conventional randomized controlled trials are impractical. By correlating genetic variants with disease risk, MR elucidates potential etiological factors and aids in the identification of new therapeutic targets. Given acne's multifactorial etiology, which includes genetic, environmental, and lifestyle components, MR facilitates a more precise delineation of these factors' causal relationships, thereby circumventing the typical confounding biases and issues of reverse causation encountered in observational studies [9].

## 2 Method

### 2.1 Research Design and Ethical Considerations

This study utilizes approved public datasets to identify potential therapeutic targets for acne. It incorporates proteomic data from Ferkingstad et al. [10], encompassing genetic associations across 4709 circulating protein levels, and acne‐specific genome‐wide association study (GWAS) data referenced from PMID: 36922633 [11]. All datasets have received ethical clearance from the pertinent institutions and adhere to all relevant privacy protection guidelines.

### 2.2 Data Sources

Proteomic data, derived from 35 559 Icelandic samples, were obtained using a multiplexed, modified oligonucleotide fluorescence labeling technique to quantify 4709 circulating protein levels and compile genetic association summary statistics. Prior to the genome‐wide association analysis, all data underwent rank‐inverse normal transformation and were standardized by sex and age. Detailed methodology is described in the original publication [10]. The acne‐related genetic association data, sourced from Teder‐Laving et al.'s study titled “Genome‐wide meta‐analysis identifies novel loci conferring risk of acne vulgaris,” analyzed 34 422 acne patients and 364 991 healthy controls from three distinct European ancestry groups [11]. These studies met rigorous case definitions and genetic data quality controls.

### 2.3 Mendelian Randomization Analysis Methods

In this research, plasma circulating proteins served as exposures with acne as the outcome. The primary analysis employed the “TwoSampleMR” tool (https://github.com/MRCIEU/TwoSampleMR) to conduct a two‐sample MR analysis, calculating each SNP's impact on protein levels and acne risk and estimating the overall influence of proteins on acne risk. Additionally, summary‐data‐based mendelian randomization (SMR) analysis utilized the SMR software tool, setting the *p* value threshold at 0.05. Heterogeneity tests (HEIDI) evaluated whether genetic tools such as SNPs affected multiple traits simultaneously, retaining results with *p* > 0.1 for further analysis. Finally, the Benjamini–Hochberg (B–H) method corrected for false discovery rate (FDR), setting it at 5%.

### 2.4 Colocalization Analysis Methods

**FIGURE 1:**
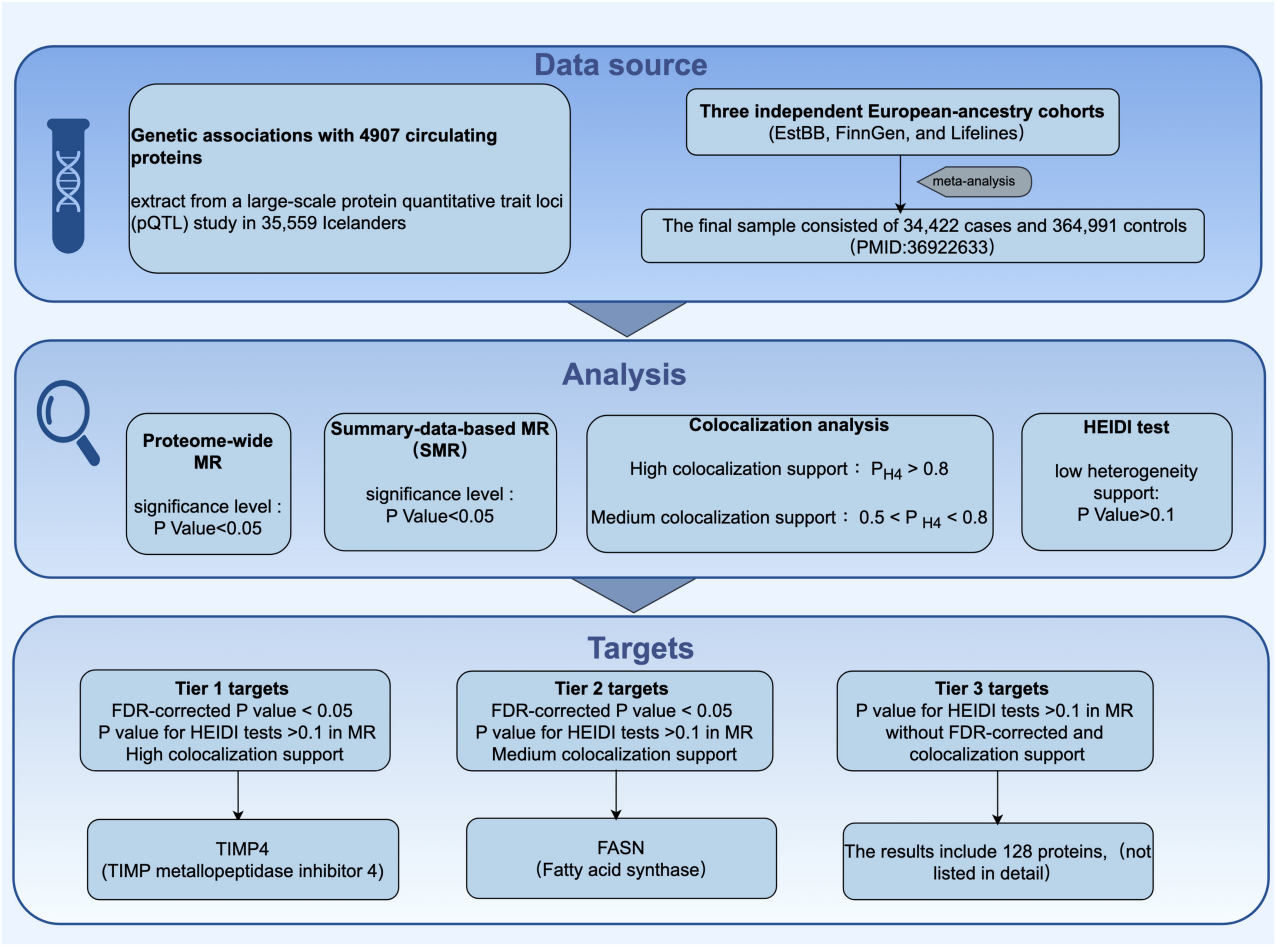
Study design. FDR, false discovery rate; HEIDI, heterogeneity in dependent instrument; MR, mendelian randomization; SMR, summary‐data‐based mendelian randomization.

Coloc colocalization analysis assesses whether two related phenotypes share common genetic factors. This process involves analyzing SNP associations with each phenotype and calculating posterior probabilities to support various hypotheses. Situations where different SNPs associate with distinct phenotypes without linkage disequilibrium may not conclusively prove shared causal variants. This study's colocalization analysis, utilizing the “coloc” R package, evaluates multiple hypotheses: H0indicates no significant association between phenotypes and any SNP within a specific genomic region; H1/H2 denotes significant association of either phenotype with SNPs in region; H3 suggests significant associations of both phenotypes with SNPs driven by different causal variants; and H4 indicates both phenotypes significantly associate with SNPs driven by the same causal variant. Posterior probabilities (PH4) < 0.8 signify strong evidence of shared causal variants, suggesting a potential causal relationship between protein levels and acne. Proteins with PH4 between 0.5 and 0.8 provide moderate colocalization support, indicating possible but inconclusive shared causal associations. These findings prioritize proteins for further analysis, advancing those with high or moderate support for validation through MR sensitivity analysis. (Figure 1).

## 3 Results

### 3.1 Genome‐Wide Protein Mendelian Randomization Analysis

In this study, we utilized SMR analysis to investigate the relationship between protein levels and acne risk, applying a *p* value threshold of 0.05 for the MR analysis. A HEIDI test was also employed to evaluate heterogeneity and minimize potential effects of pleiotropy. Initially, 128 proteins were identified as significantly associated with acne risk. Following the application of the Benjamini–Hochberg (B–H) method for FDR correction set at 0.05, only two proteins, FASN fatty acid synthase (FASN) and tissue inhibitor of metalloproteinases 4 (TIMP4), remained significantly associated with acne risk. Detailed results are documented in Table 1. For FASN, a negative *β*‐value (*β* = −0.264) suggests that increased genetically predicted levels of FASN correlate with a lower risk of acne (OR = 0.768, 95% CI: 0.676–0.872, *p* = 4.685E‐05), a finding that persisted after FDR correction (*p* = 0.039). Furthermore, the HEIDI test yielded a *p* value of 0.037, confirming the robustness of this result against pleiotropy. Conversely, TIMP4 exhibited a positive *β*‐value of 0.157, linking higher genetically predicted levels of TIMP4 to an increased acne risk (OR = 1.169, 95% CI: 1.103–1.241, *p* = 1.956E‐07). Despite the HEIDI test *p* value of 0.055 being slightly above the conventional threshold, the association remained significant post‐FDR correction (*p* = 3.33E‐04), indicating that the relationship between TIMP4 and acne risk is not driven by pleiotropy.

**TABLE 1:** Mendelian randomization analysis.

Gene	Beta	SE	OR (95% CI)	*p*	*p* (HEIDI test)	*p* after FDR adjustment	Number of SNPs used in HEIDI test
FASN	−0.264	0.065	0.768 (0.676–0.872)	4.685E‐05	0.037	3.99E‐02	20
TIMP4	0.157	0.030	1.169 (1.103–1.241)	1.956E‐07	0.055	3.33E‐04	20

Abbreviations: CI, confidence interval; FDR, false discovery rate; HEIDI, heterogeneity in dependent instruments; OR, odds ratio.

### 3.2 Colocalization Analysis

**FIGURE 2:**
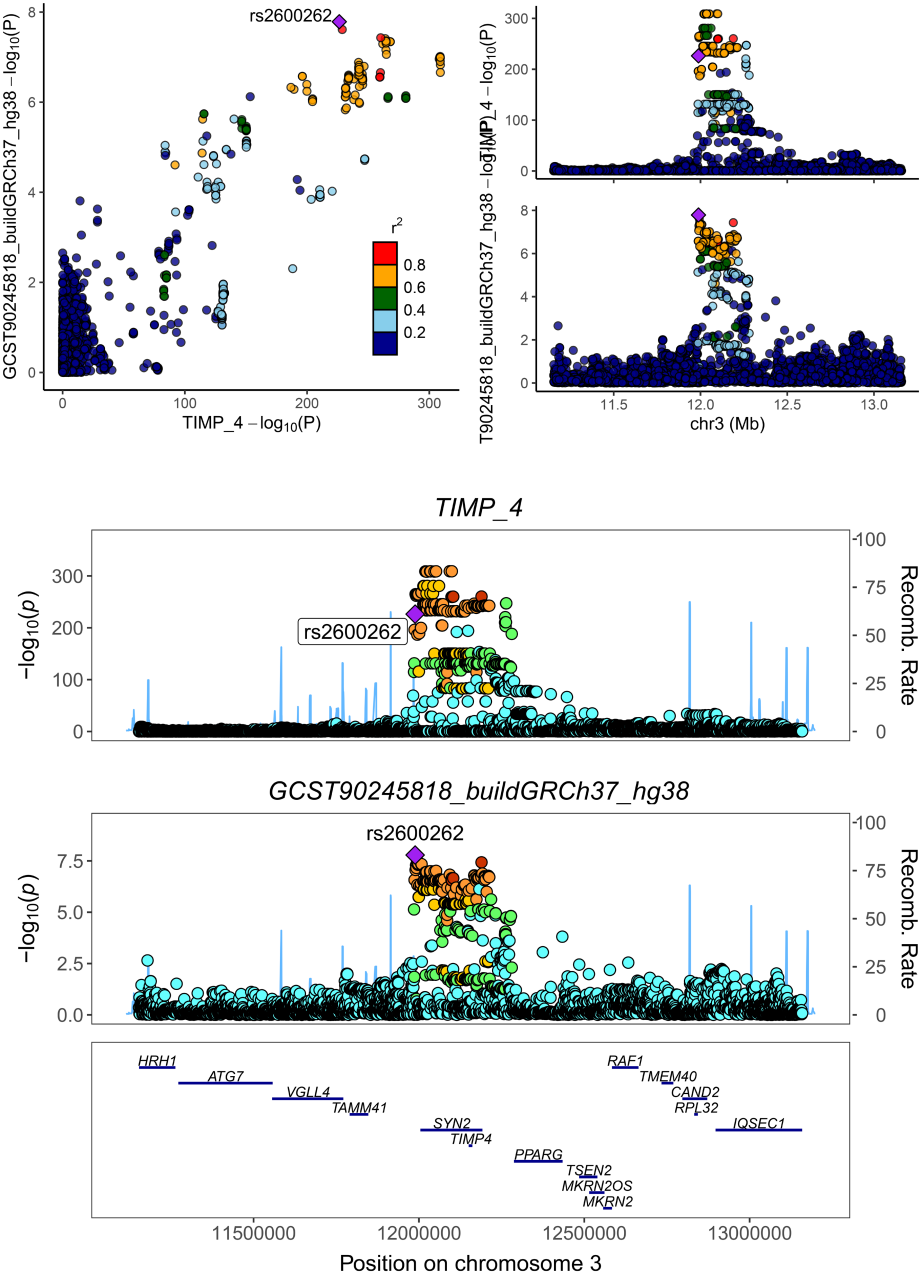
Colocalization of circulating proteins with acne.

Colocalization analysis assessed the genetic correlations between circulating proteins and acne risk. Initial analysis focused on TIMP4 and FASN, using GWAS data post‐FDR correction, as detailed in Table 2. The colocalization results indicated a PH4 (probability of sharing the same causal variant) of 0.9323 for TIMP4, suggesting a high likelihood of sharing a common causal variant with acne risk. Similarly, the PH4 for FASN was 0.8905, also denoting a high probability of a shared causal variant, underscoring the genetic link between these proteins and acne. Further colocalization analysis from a proteomics and biological mechanisms perspective reinforced the high probability of TIMP4 sharing a causal variant with acne risk, as depicted in Figure 2. Although similar results were not observed for FASN, the genetic associations visualized in the TIMP4 gene region, particularly at rs2600262, indicated significant associations (−log_10_(P)), confirming the genetic linkage. Recombination rate analysis provided insights into the genetic landscape near rs2600262, identifying potential connections to acne risk. This dynamic representation of genetic variations offers critical insights into genetic regulatory mechanisms. Our analysis also spotlighted several other genes implicated in acne risk, including HRH1, ATG7, and VGLL4.

**TABLE 2:** Colocalization of circulating proteins with acne.

Outcome	Proteins	P_H0_	P_H1_	P_H2_	P_H3_	P_H4_
Acne	TIMP4	1.760E‐19	2.095E‐08	5.766E‐13	6.772E‐02	9.323E‐01
	FASN	1.049E‐49	3.317E‐14	3.492E‐37	1.095E‐01	8.905E‐01

## 4 Discussion

### 4.1 Acne: Etiology and Implications

Acne is a chronic inflammatory disease of the hair follicle sebaceous gland unit, caused by increased sebum production induced by androgens, keratinization changes, inflammation, and colonization of Propionibacterium acnes in the hair follicles of the face, neck, chest, and back [1, 12]. Among these factors, the activity of the sebaceous glands and the inflammatory response play key roles in the development of acne. The sebum produced by the sebaceous glands is a complex mixture of lipids that is crucial for maintaining the skin barrier function [13]. Excessive sebum combined with abnormal proliferation of keratinocytes leads to follicular obstruction and the formation of comedones, which are early manifestations of acne. Moreover, the disturbance in the composition and content of sebum is closely related to the severity of acne. Additionally, when the sebaceous glands produce excess sebum, the microenvironment of the skin changes, providing conditions for the proliferation of microbes such as Propionibacterium acnes. The proliferation of these microbes not only directly stimulates inflammation in the skin but can also activate the skin's immune system by releasing metabolic products and cytokines. In particular, P. acnes can activate toll‐like receptors (TLRs), triggering the release of inflammatory cytokines and thus initiating the immune inflammatory response of acne [14, 15, 16]. Overall, the pathogenesis of acne is the result of multifactorial interactions.

### 4.2 Role of Tissue Inhibitor of Metalloproteinases 4 in Acne

TIMP4 is an important inhibitor of matrix metalloproteinases (MMPs), which regulates the degradation of the extracellular matrix (ECM), thereby affecting cell proliferation, migration, and apoptosis [17]. Recent studies have revealed that TIMPs also play a significant role in skin diseases [18], including TIMP4, which is the focus of this article. Existing research suggests that TIMP4 may be associated with the risk of acne by influencing sebaceous gland activity, inflammatory responses, tissue remodeling, and ECM degradation. Overactive sebaceous glands are one of the key factors in the onset of acne [19]. TIMPs regulate the activity of MMPs and may indirectly affect the function of sebaceous glands. MMPs play a crucial role in maintaining the structure and function of skin tissue, including the remodeling of the ECM surrounding the sebaceous glands. Overexpression of TIMPs may inhibit the activity of MMPs, thereby affecting the normal keratinization process of sebaceous gland ducts and promoting the formation of acne [20, 21]. Moreover, by inhibiting the activity of MMPs, TIMPs could reduce the inflammatory responses caused by the overactivity of MMPs. Studies have shown that TIMPs can inhibit metalloproteinases involved in inflammation and tissue damage, such as MMP‐9, thereby reducing the infiltration of inflammatory cells and the release of inflammatory mediators [22]. This mechanism suggests that TIMP4 could not only reduce the severity of acne through its protective role as a physical barrier but also potentially lower the recurrence rate of acne by regulating immune responses. Additionally, TIMP4 is involved in regulating tissue remodeling and maintaining the integrity of the ECM structure. In acne patients, excessive degradation or improper remodeling of the ECM may lead to the destruction of structures around hair follicles, promoting the formation of acne lesions [23].

Current research indicates that the expression of TIMP4 is associated with the development of various skin diseases. A study on the risk of acne scarring in the Chinese Han population related to the TIMP2 gene mutation rs4789932 suggests that changes in the expression of this gene may disrupt the TIMP/MMP balance, leading to the formation of acne scars [24]. Further research has found that the TIMP‐2 (−418 G/C) polymorphism is distributed differently in acne patients compared with controls, suggesting that the TIMP‐2 genotype may disrupt the balance between MMPs and TIMPs, increasing the tendency to develop acne [25]. Although the focus of the research is on TIMP2, it highlights the importance of the TIMP family in the pathogenesis of acne. More specifically, studies indicate that TIMP4 is present in the facial sebum of acne patients and that a decrease in TIMP4 expression following treatment with isotretinoin is associated with clinical improvement in acne. This suggests that TIMP4 in the sebum may be involved in the pathological process of acne, including its protective role on the ECM in scenarios of inflammation and abnormal keratinization [26].

### 4.3 Role of Fatty Acid Synthase in Acne

Fatty acid synthase is an enzyme that plays a crucial role in the fatty acid synthesis pathway, responsible for catalyzing the synthesis of long‐chain fatty acids [27]. Similar to TIMP4, FASN may also influence the risk of acne onset through pathways such as sebaceous gland activity and inflammatory responses. FASN is the key enzyme in sebaceous glands catalyzing the production of fatty acids, which are one of the main components of sebum. Sebum is an oily substance, and its excessive production and clogging of pores can lead to the formation of acne. By inhibiting FASN, the production of sebum can be reduced, thereby decreasing the likelihood of follicular blockage and inflammation. Additionally, FASN inhibitors, by reducing sebum production, might indirectly reduce inflammation. Since acne lesions are often associated with inflammatory responses, controlling excessive sebum production can alleviate this inflammation. Furthermore, FASN may also participate in regulating inflammatory responses by affecting cell signaling pathways [28, 29]. For example, FASN can regulate the expression of inflammatory cytokines through the nuclear factor‐kappa B (NF‐κB) pathway, thereby participating in the inflammatory responses in the pathogenesis of acne [30]. In recent years, as the understanding of FASN's biological functions and pathological roles has deepened primarily in the context of cancer, it has been found that FASN is overexpressed in many types of cancer and is closely related to tumor cell growth, survival, and metabolic reprogramming [31, 32]. Existing studies suggest that FASN inhibitors also hold great potential in treating acne. According to a public report at the 2024 American Academy of Dermatology (AAD) annual meeting, ASC40, a Fatty Acid Synthase Inhibitor for treating acne, has completed preliminary clinical trials, showing that 10 days of ASC40 treatment can significantly reduce facial sebum palmitic acid levels. However, as Fatty Acid Synthase Inhibitors have not yet been formally recognized as an acne treatment medication and have not entered widespread clinical use, further research and observation are needed to evaluate their long‐term safety and efficacy.

### 4.4 Connections Between Other Relevant Proteins and Acne

Our research, through colocalization analysis, has identified genes such as HRH1, ATG7, and VGLL4 as related to the risk of acne, but currently, the clinical applications of these genes or proteins are mainly still in the research phase. Research on HRH1 inhibitors primarily focuses on their anti‐inflammatory effects [33], while potential treatment strategies for ATG7 and VGLL4 are more concerned with their ability to regulate cell functions [34, 35]. Continued clinical trials and research will help determine the practical application value of these targets in the treatment of acne.

### 4.5 Strengths and Limitations of This Study

In the research exploring potential therapeutic targets for acne, through Mendelian randomization (MR) analysis and colocalization analysis, we identified two proteins significantly associated with acne risk: FASN and TIMP4. Through this method, we can not only reveal the potential causal relationship between proteins and acne risk but also identify possible therapeutic targets, providing new biological insights into the treatment of acne and potentially guiding future clinical intervention strategies. This study not only demonstrates significant advantages in methodological innovation and transparency in data handling but also faces limitations such as data homogeneity and constraints in causal inference. Although the dataset used is extensive and public, it is mainly focused on specific populations (such as Icelanders), which may limit the general applicability of the findings. Future studies need to be validated in more diverse populations to ensure the broad applicability and interpretative power of the research results. Additionally, despite the rigorous design of the study, relying on public datasets may introduce inherent biases from the original data collection process, such as selection bias and information bias. Future research should reduce this risk through multicenter, multi‐sample designs. Moreover, while MR can reduce the impact of confounding factors and improve the reliability of causal inference, there remains the issue of horizontal heterogeneity that cannot be completely eliminated. For example, a single SNP may affect multiple proteins, not just the specific protein of interest. This study has identified target proteins with a causal relationship to the risk of acne through MR analysis, but the mechanisms of action and applications of these proteins still need further verification in subsequent experiments.

## Conflicts of Interest

The authors declare no conflicts of interest. All authors have read and approved this version of the article, and due care has been taken to ensure the integrity of the work. Neither the entire paper nor any part of its content has been published or has been accepted elsewhere. It is not being submitted to any other journal.

## Data Availability

The proteomic data were obtained from Ferkingstad et al. (2021) (doi: 10.1038/s41588‐021‐00978‐w), and are available in the European Genome‐phenome Archive (EGA) at https://ega‐archive.org under accession number EGAS00001002555. The acne‐related genome‐wide association study (GWAS) data were sourced from Teder‐Laving et al. (2023) (doi: 10.1038/s41431‐023‐01326‐8), available in the GWAS Catalog at https://www.ebi.ac.uk/gwas/ under accession number GCST90038690. These datasets have been ethically approved for use and are accessible following the necessary guidelines provided by the respective repositories. The data that support the findings of this study are available on request from the corresponding author. The data are not publicly available due to privacy or ethical restrictions.
